# Wastewater-based intestinal protozoa monitoring in Shanghai, China

**DOI:** 10.1128/spectrum.04032-23

**Published:** 2024-09-24

**Authors:** Yanyan Jiang, Zhongying Yuan, Yaxue Wang, Jing Zhang, Yujuan Shen, Jianping Cao

**Affiliations:** 1 National Key Laboratory of Intelligent Tracking and Forecasting for Infectious Diseases, National Institute of Parasitic Diseases at Chinese Center for Disease Control and Prevention, Chinese Center for Tropical Diseases Research, NHC Key Laboratory of Parasite and Vector Biology, WHO Collaborating Centre for Tropical Diseases, Shanghai, China; 2 School of Global Health, Chinese Center for Tropical Diseases Research, Shanghai Jiao Tong University School of Medicine, Shanghai, China; Hubei University of Medicine, Shiyan city, Hubei Province, China

**Keywords:** *Cryptosporidium *spp., *Giardia duodenalis*, *Enterocytozoon bieneusi*, influent wastewater, effluent wastewater, intestinal protozoa, microbial source tracking

## Abstract

Intestinal protozoa *Cryptosporidium* spp., *Giardia duodenalis*, and *Enterocytozoon bieneusi* have been implicated in serious waterborne outbreaks worldwide. Wastewater-based epidemiology (WBE) is a promising approach for evaluating the disease prevalence in a catchment population in that it monitors the contamination level of the intestinal pathogens in wastewater. We collected 48 urban wastewater samples (24 from influents and 24 from effluents) from the Yangpu Wastewater Treatment Plant (YPWTP) in Shanghai, China. We identified *Cryptosporidium* spp., *G. duodenalis*, and *E. bieneusi* by nested polymerase chain reaction (PCR) amplification. *Cryptosporidium hominis* and subtype IdA14 were identified in two samples by analyzing the sequences of small subunit ribosomal RNA (SSU rRNA) and 60–kDa glycoprotein (*gp60*) genes, respectively. The *G. duodenalis* sub-assemblage AII (*n* = 8) and assemblage C (*n* = 4) in 12 samples were determined by analyzing triosephosphate isomerase (*tpi*) gene sequences. The *E. bieneusi* genotype A was identified in one sample by analyzing the sequence of the internal transcribed spacer (ITS) region of the rRNA gene. These findings suggest that improving wastewater treatment and monitoring the virility of pathogens in effluents is critical. We observed similar prevalence and genotypes/subtypes of the three intestinal protozoa in our wastewater samples as those reported in previous studies, providing evidence that WBE can be used as an effective epidemic management tool.

**IMPORTANCE:**

*Cryptosporidium* spp., *Giardia duodenalis,* and *Enterocytozoon bieneusi* are common intestinal protozoa causing diarrhea. The infective oocysts, cysts, and spores released in feces can survive in different environments, including multiple types of water bodies. Humans can acquire these intestinal protozoan infections *via* the fecal-oral route as in waterborne transmission. Wastewater-based epidemiology can rapidly and reliably detect and monitor the emergence and spread of waterborne diseases. We detected *Cryptosporidium* spp., *G. duodenalis,* and *E. bieneusi* in a wastewater treatment plant in Shanghai, China, reflecting the occurrence and genetic characterizations of the three intestinal pathogens from community members served by the wastewater treatment plant.

## INTRODUCTION

*Cryptosporidium* spp., *Giardia duodenalis*, and *Enterocytozoon bieneusi* are common intestinal protozoa responsible for diarrhea in humans worldwide (1–3), especially in developing countries (4). *Cryptosporidium* spp. have been detected in at least 260 animal species, *G. duodenalis* in 40, and *E. bieneusi* in 210, suggesting their zoonotic nature (5–7). Humans acquire infections through the consumption of food and water contaminated by *Cryptosporidium* oocysts, *G. duodenalis* cysts, and *E. bieneusi* spores released in the feces of the host organism. These oocysts, cysts, or spores are immediately infectious after defecation and they survive in a variety of environments, such as bodies of water, soil, and food products (8, 9).

The epidemiological role of water in parasitic disease transmission is well recognized. Water-related outbreaks of cryptosporidiosis (*n* > 524) (10), giardiasis (*n* > 344) (11), and microsporidiosis from *E. bieneusi* (at least one event) (12) have been documented. These pathogens are listed on the United States Environmental Protection Agency (EPA) microbial contaminant candidate list of concern for waterborne transmission (https://www.epa.gov/ground-water-and-drinking-water/national-primary-drinking-water-regulations).

Wastewater treatment plants mainly process domestic wastewater from humans. Human health biomarkers can be excreted *via* feces and urine and end up in wastewater. *Cryptosporidium* spp., *G. duodenalis,* and *E. bieneusi* have been detected in wastewater in wastewater treatment plants worldwide (13, 14). Wastewater-based epidemiology (WBE) is an approach that monitors the pathogen levels in wastewater concurrent with the disease prevalence in communities. Treated wastewater is discharged into rivers, and used downstream for drinking water, irrigation, and recreation, potentially causing environmental contamination or disease outbreaks. Improving the monitoring of pathogens in wastewater samples in wastewater treatment plants is essential. Currently, microscopy, immunology, and nucleic acid-based tools (mainly the PCR method) have been employed for the detection and identification of pathogens in water samples (15, 16). However, only PCR-based methods accurately identify the pathogens to the species/genotype and subtype levels, thereby tracing the source of infection or contamination (17).

In China, *Cryptosporidium* spp., *G. duodenalis,* and *E. bieneusi* have been detected in various water bodies (18–32) (Table 1). Ten studies reported their presence in wastewater in nine cities, with occurrence rates of 4.0%–100% for *Cryptosporidium* spp (20, 21), 19.5%–100% for *G. duodenalis* (21, 30), and 42.6%–100% for *E. bieneusi* (25, 31). Shanghai is one of the biggest cities in China, with a population of ~24 million. However, human epidemiological investigations of *Cryptosporidium* spp., *G. duodenalis,* and *E. bieneusi* have been limited to a few vulnerable populations, including children (33–35), diarrheal patients (4, 36, 37), and HIV/AIDS patients (38). The infectivity of each pathogen within local populations is undetermined. Since the surveillance of wastewater samples in urban wastewater treatment plants can indicate the prevalence and trends of intestinal pathogens in humans in designated areas, we carried out a 1-year molecular surveillance study to determine the occurrence rate and assess the possible source of infection at the genotype and subtype levels of *Cryptosporidium* spp., *G. duodenalis,* and *E. bieneusi* in wastewater from a treatment plant in Shanghai.

**TABLE 1:** Distribution of *Cryptosporidium* spp., *G. duodenalis,* and *E. bieneusi* in water bodies by city in China^*a*^

Types of water bodies	Sampling period	Location	Protozoan	Rate (positive no./examined no.)	Genotype/subtype (n)	Reference
Swimming pool	August 2015	Beijing	*Cryptosporidium*	21.2 (7/33)	*C. hominis* (6); *C. parvum* (1)	(18)
			*G. duodenalis*	12.1 (4/33)	A (3); B (1)	
	June–October 2017	Tianjin	*Cryptosporidium*	82.7 (43/52)	*C. parvum* (3); *C. andersoni* (3); *C. hominis* (1); *C. meleagridis* (1); *C. fragile* (1); *C. ubiquitum* (1)	(19)
			*G. duodenalis*	98.1 (51/52)	A (3); B (1); D (1)	
River/lake water	March–September 2011	The three Gorges Reservoir	*Cryptosporidium*	85.3 (52/61)	*C. andersoni* (6); *C. suis* (2); *C. hominis* (1); *C. bovis* (1); *C. hominis + C. baileyi* (1); *C. bovis + C. suis* (2); *C. hominis + C. suis* (1)	(20)
			*G. duodenalis*	60.7 (37/61)	A (5); E (1 + 1); A + B (2); A + E (1)	
	2015–2017	Qinghai Tibetan plateau area	*Cryptosporidium*	2.4 (3/127)	*C. hominis* (2); *C. andersoni* (1)	(21)
			*G. duodenalis*	20.5 (26/127)	A (26)	
	March 2013–March 2014	Shanghai	*Cryptosporidium*	37.6 (67/178)	*C. andersoni* (38); *C. suis* (27); *C. baileyi* (16); *C. scrofarum*(8); *C. meleagrid*is (4); *C. parvum* (3); *C. hominis* (2); *C. ryanae* (1); *C. cuniculus* (1); *C. fragile* (1); rat genotype IV (1); avian genotype II (1); avian genotype III (1)	(22)
			*E. bieneusi*	31.5 (56/178)	EbpC (37), EbpA (7), D (7), CS-8 (6), PtEb IX (4), Peru 8 (1), Peru 11 (1), PigEBITS4 (1), EbpB (1), G (1), O (1), RWSH1 (1), RWSH2 (1), RWSH3 (1), RWSH4 (1), RWSH5 (1), RWSH6 (1)	
	June, August, October 2009	Tongxiang	*Cryptosporidium*	78.7 (37/47)	*C. suis* (2); *C. fragile* (1); avian III (2); pig II (1); *C. ubiqiutum* (1)	(23)
Source water^*b*^	May 2009–January 2010	Shanghai	*Cryptosporidium*	40.0 (20/50)	*C. anderson*i (7); *C. suis* (2); *C. hominis* (1); *C. anderson*i + *C. baileyi* (1); *C. anderson*i + *C. baileyi + C. meleagridis* (1); *C. anderson*i + *C. suis* (8)	(24)
Slaughter water	2015–2017	Qinghai Tibetan plateau area	*Cryptosporidium*	0 (0/153)	–	(21)
			*G. duodenalis*	11.1 (17/153)	A (16); E (1)	
Wastewater	June–September 2012, June–September 2014	Shanghai^*c*^	*Cryptosporidium*	30.0 (12/40)	*C. hominis* (5); *C. viatorum* (4); *C. ubiquitum* (4); *C. parvum* (2); *C. meleagridis* (3); *C. baileyi* (1); *C. muris* (2)	(25)
			*G. duodenalis*	82.5 (33/40)	AII (28); AII-like (2); G (1); A (2)	
			*E. bieneusi*	92.5 (37/40)	D (17); Peru11 (1); D + PigEBITS7 (7); D + SHW1 (3); D + Henan V (7); D + Peru8 + Type IV (1); D + Peru8 + Peru11 + Henan V (1)	
	August–October 2018	Guangzhou^*d*^	*Cryptosporidium*	14.1 (46/326)	Rat genotype IV (14); *C. muris* (12); *C. baileyi* (9); *C. felis* (7); *C. parvum* (4); *C. bovis* (4); *C. occultus* (3); *C. meleagridis* (2); *C. serpentis* (1); *C. canis* (1); *rat genotype I* (1)	(26)
			*G. duodenalis*	49.4 (161/326)	A (132); B (8); C (2); F (2), A + B (2)	
			*E. bieneusi*	48.2 (157/326)	D (88); Type IV (35); Peru8 (10); Type IV + D (9); PtEb IX (3); EbpC (3); Peru11 (2); Type IV + Peru11 (1); Peru6 (1); MWC-m1 (1); GZW1 (1); GZW2 (1); GZW3 (1); Peru8 + Type IV (1)	
	April 2009–March 2010	Harbin^*d*^	*Cryptosporidium*	31.3 (15/48)	*C. andersoni* (14); *C. ubiquitum* (1)	(27)
			*G. duodenalis*	50.0 (24/48)	AII (18); B (6)	
	July 2007–May 2009	Nanjing^*d*^	*Cryptosporidium*	36.8 (32/87)	*C. hominis* (6); *C. suis* (7); pig genotype II (1); *C. cuiculus* (3); VaA31 (1); *C. meleagridis* (2); *C. baileyi* (6); *C. muris* (18); rat genotype I (1)	(28)
			*G. duodenalis*	67.8 (59/87)	AII (38); B3 (1); B4 (1); AII + B2 (2); AII + B3 (3); AII + B4 (1); AII + B6 (1); AII + B8 (1); AII + B10 (1); AII + B12 (1); AII + B14 (1); AII + B (6)	
			*E. bieneusi*	62.1 (54/87)	D (50); Peru11 (2); Peru6 (1); Peru8 (1); WL14 (2); WL12 (1)	
	April–July 2008	Qingdao^*d*^	*Cryptosporidium*	68.8 (75/109)	*C. hominis* (1); *C. parvum* (2); *C. canis* (1); *C. suis* (6); *C. meleagridis* (7); *C. bailey*i (2); *C. muris* (2); rat genotype I (2); rat genotype IV (8)	
			*G. duodenalis*	97.3 (106/109)	AII (97); B2 (1); B5 (1); AII + B13 (1); AII + B15 (1); AII + B16 (1)	
			*E. bieneusi*	91.7 (100/109)	D (71); Type IV (5); EbpC (1); EbpD (1); Peru6 (3); PtEb IV (1); BEB6 (1); PtEb IX (4); PigEBITS7 (1); WW3 (15); WW6 (4); WW1 (4); WW8 (4); WW4 (3); WW2 (1); WL4 (1); WW5 (1); WW7 (1); WW9 (1)	
	2015–2017	Qinghai Tibetan plateau area^*d*^	*Cryptosporidium*	4.0(7/176)	*C. hominis* (3); *C. parvum* (1); *C. struthionis* (1); *C. canis* (1); unknown (1)	(21)
			*G. duodenalis*	30.7 (54/176)	A (54)	
	December 2006–Apr 2007	Shanghai^*d*^	*Cryptosporidium*	70.0 (63/90)	*C. homin*is (47); *C. meleagridi*s (5); *C. parvum* (1); *C. hominis + C. baileyi* (1); *C. hominis + C. meleagri*dis (1); *C. hominis* + new genotype (1); *C. hominis + C. baileyi* (1); *C. hominis + C. muris* (1); *C. hominis + C. baileyi* (1); *C. hominis + C. meleagridis* (1); *C. hominis + C. suis* (1); *C. hominis* + rat genotype (1); rat genotype +avian genotype III (1)	(29)
	June–September 2012, June–September 2014			37.5 (15/40)	*C. hominis* (4); *C. viatorum* (3); *C.parvum* (4); *C. felis* (1); *C. baileyi* (3); *C. meleagridis* (1); *C. ubiquitum* (5); *C. muri*s (3); rat genotype I (1); rat genotype IV (1)	(25)
	March–November 2009			18.9 (31/164)	*C. hominis* (17); *C. meleagridis* (7); *C. parvum* (1); *C. hominis + C. meleagridis* (1); *C. hominis + C. parvum + C. meleagridis* (5)	(30)
	December 2006–April 2007		*G. duodenalis*	94.4 (85/90)	AII (51); B1 (1); AII + B1 (1); AII + B (30)	(28)
	June–September 2012, June–September 2014			80.0 (32/40)	AII (30); A (2)	(25)
				19.5 (32/164)	AII (26); B (6)	(30)
	December 2006–April 2007		*E. bieneusi*	94.4 (85/90)	D (78); PigEBITS7 (6); Type IV (4); EbpC (2); Peru11 (2); Peru8 (1); PigEBITS8 (1)	(28)
	June–September 2012, June–September 2014			100.0 (40/40)	D (20); PigEBITS7 (1); Type IV (1); SHW2 (1); D + PigEBITS7 (5); D + SHW1 (1); D + SHW1 (1); D + SHW2 (1); D + Peru8 (1); D + Peru11 (1); D + Henan V (7); D + PigEBITS7 + SHW1 (1)	(25)
	March–November 2009			74.4 (122/164)	D (97); Peru 11 (5); EbpC (5); Henan V (3); PigEBITS7 (2); Peru 8 (1); SHW3 (1); SHW4 (1); SHW5 (1); SHW6 (1); SHW7 (1); D + Peru 11 (2); D + Peru 11 (2); D + Peru 8 (1); D + SHW4 (1)	(30)
	April–July 2008	Wuhan^*d*^	*Cryptosporidium*	47.0 (47/100)	*C. hominis* (19); *C. andersoni* (4); *C. canis* (1); *C. suis* (17); Pig genotype II (5); *C. cuniculus* (2); *C. meleagridis* (6); *C. baileyi* (4); *C. muris* (10); rat genotype I (1); rat genotype IV (2)	(28)
			*G. duodenalis*	69.0 (69/100)	AII (57); AII + B7 (1); AII + B9 (1); AII + B11 (1)	
			*E. bieneusi*	99.0 (99/100)	D (80); Peru8 (11); Peru11 (6); EbpC (5); WW3 (5); PtEb IX (3); Type IV (3); Peru6 (1); C (1); EbpA (2); BEB6 (1); WL4 (1)	
	November 2014–October 2015	Zhengzhou^*d*^	*E. bieneusi*	42.6 (46/108)	BEB6 (13); D (8); EbpA (4); I (1); J (1); PigEBITS5 (4); HNWW2 (3); HNWW1 (2); HNWW4 (2); PigEbIX (2); Peru6 (2); HNWW3 (1); HNWW5 (1); Peru8 (1); Type IV (1)	(31)
	March–September 2011	The three Gorges Reservoir^*e*^	*Cryptosporidium*	100.0 (5/5)	*C. hominis* (1); *C. meleagridis* (1); *C. hominis + C. andersoni* (2); *C. andersoni* (1)	(20)
			*G. duodenalis*	100.0 (5/5)	A (2); B (2); A + B (1)	
	September 2014–March 2015	Shanghai^*e*^	*Cryptosporidium*	40.0 (20/50)	*C. muris* (13); *C. meleagridis* (6); *C. suis*-like (3); *C. parvum* (3), rat genotype I (2); *C. hominis* (1); *C. canis* (1); *C. baileyi* (1); *C. felis* (1); rat genotype IV (1),	(32)
			*G. duodenalis*	100.0 (50/50)	AII (44); AII + A/B (unknown)^*f*^	
			*E. bieneusi*	70.0 (35/50)	D (31); ESH-03 (6); EbpC (2); ESH-04 (2); PigEBITS7 (2); EbpA (1); Peru 8 (1); Peru 11 (1); ESH-01 (1); ESH-02(1); ESH-05 (1)	

^*a*^ The bars denote negative results.

^*b*^ The source water from the Huangpu River was collected at the intake of the drinking water treatment plants for Shanghai.

^*c*^ The wastewater specimens were from the Sewer distribution system.

^*d*^ The wastewater specimens were influents from the WWTPs/sewage factory.

^*e*^ The wastewater specimens were effluents from the WWTPs.

^*f*^ *G. duodenalis* assemblages were identified based on multilocus sequence analysis (at the tpi, gdh, and *β*-giardin loci).

## RESULTS

### Protozoal contamination in wastewater

Of the 48 wastewater samples, 4.2% (2/48), 25.0% (12/48), and 2.1% (1/48) were positive for *Cryptosporidium* spp., *G. duodenalis,* and *E. bieneusi*, respectively. *Cryptosporidium* spp. and *G. duodenalis* were identified in influents and effluents, with occurrence rates of 4.2% (1/24) and 4.2% (1/24) for *Cryptosporidium* spp. and 33.3% (8/24) and 16.7% (4/24) for *G. duodenalis. E. bieneusi* was only detected in one effluent sample (4.2%, 1/24) (Table 2).

**TABLE 2:** Occurrence of *Cryptosporidium* spp., *G. duodenalis*, and *E. bieneusi* in influent and effluent samples from YPWTP in different months at genotype and subtype levels^*a*^^,^^*b*^

Type of water	Sampling time	*Cryptosporidium* spp.	*G. duodenalis*	*E. bieneusi*
		Species (n)/subtype (n)	Assemblage/sub-assemblage (n)	Genotype (n)
Influent	2011/07/15	－	AII (1)	－
	2011/08/15	－	AII (1)	－
	2011/08/30	－	C (1)	－
	2011/09/15	－	C (1)	－
	2011/09/30	－	AII (1)	－
	2012/04/15	*C. hominis* (1): IdA14 (1)	－	－
	2012/05/15	－	AII (1)	－
	2012/06/15	－	AII (1)	－
	2012/06/30	－	AII (1)	－
Effluent	2011/08/15	－	C (1)	－
	2011/08/30	－	C (1)	－
	2011/09/15	－	－	A (1)
	2011/10/15	－	AII (1)	－
	2012/05/15	－	AII (1)	－
	2012/05/30	*C. hominis* (1): IdA14 (1)	－	－
Rate		4.2% (2/48)	25.0% (12/48)	2.1% (1/48)

^*a*^ The bar denotes negative results.

^*b*^ Sampling time of only positive samples was presented in this table.

### Genotyping and subtyping of protozoa

*C. homini*s was the species identified in two of the *Cryptosporidium*-positive samples. These samples had six base differences between them (KJ019853 and KJ019854) within the small subunit ribosomal RNA (rRNA) gene. The former exhibited 100% similarity to a human-derived sequence (AY204230) from England, while the latter showed a match with a human-derived sequence (MK982462) from Bangladesh. Both of them produced the same 60-kDa glycoprotein (*gp60*) gene sequence (KJ019855), and had 100% similarity to the sequence of subtype IdA14 (GU214350) in the United Kingdom.

Sequence analysis of the triosephosphate isomerase (*tpi*) gene of 12 *G*. *duodenalis*-positive samples identified sub-assemblage AII (*n* = 8) and assemblage C (*n* = 4). Six and two samples of the sub-assemblage AII had 100% similarity with the human-derived sequences of sub-assemblage AII (OM115972) and AII (MH673809), respectively. Four assemblage C samples produced two different obtained sequences that were identical to a dog-derived sequence (KP258397) and a raccoon dog-derived sequence (KX014799).

Sequence analysis of the internal transcribed spacer (ITS) region (243 bp) of the rRNA gene identified known genotype A (*n* = 1) of *E. bieneusi*.

## DISCUSSION

The surveillance of wastewater in municipal wastewater treatment plants is useful for understanding intestinal diseases. We detected *Cryptosporidium* spp. (4.2%), *G. duodenalis* (25.0%), and *E. bieneusi* (2.1%) in wastewater samples at the Yangpu Wastewater Treatment Plant (YPWTP) in Shanghai, China. This suggests that locals may have had infections caused by these pathogens. This was consistent with the results of previous epidemiological studies involving these pathogens in the investigated area (4, 33–38) (Table 3). To date, 8,732, 6,588, and 2,625 people in Shanghai have participated in epidemiological investigations of *Cryptosporidium* spp., *G. duodenalis,* and *E. bieneusi*, respectively. Within these study populations, 2.3%, 3.9%, and 2.0% were diagnosed as having an infection of *Cryptosporidium* spp., *G. duodenalis,* and *E. bieneus*i, respectively. Wastewater surveillance can be used as an indicator for predicting intestinal disease incidence. Continuous wastewater monitoring can reveal trends, including the emergence of new intestinal pathogens and/or marked prevalence increases in known pathogens. We noted that the three intestinal pathogens were all detected in effluent wastewater samples. Treated wastewater that is discharged into the Huangpu River is used downstream for drinking, irrigation, and recreation. If these pathogens survive, downstream inhabitants have an increased risk of infection from eating uncooked vegetables, drinking the water, and swimming. Monitoring and eliminating the pathogens in the wastewater treatment process can reduce the risk of contamination of environments and infection of inhabitants.

**TABLE 3:** Prevalence of *Cryptosporidium* spp., *G. duodenalis,* and *E. bieneusi* based on molecular detection in three populations in Shanghai^*a*^^,^*^b^*

Population	Sampling period	*Cryptosporidium* spp.		*G. duodenalis*		*E. bieneusi*		Reference
		Positive no. /examined no. (%)	Species (n): subtype (n)	Positive no. /examined no. (%)	Assemblage (n)	Positive no. /examined no. (%)	Genotype (n)	
Children	September 2007–October 2009	102/6,284 (1.6)	*C. hominis* (92): IaA14R4 (36), IdA19 (37); IbA19G2 (1); IdA14 (1); IaA18R4 (1); IgA14 (1) *C. meleagridis* (6): NA *C. canis* (2): NA *C. felis* (2): NA	–	–	–	–	(33)
		–	–	40/4045 (1.0)	AII (25); B (11)	24/573 (4.2)	Peru 11 (6); EbpA (2); SH2 (3); D, EbpC, SH1, and SH3–12 (one each)	(34)
	December 2011, June 2012, September2013	39/396 (9.9)	*C. hominis* (39): IaA14R4 (26)	161/396 (40.7)	AII (153)	–	–	(35)
Diarrhea patients	October 2012–March 2013	34/252 (13.5)	*C. andersoni* (34)	34/252 (13.5)	B (1); C (16)	17/252 (6.8)	–	(36)
	May–July 2014	–	–	1/95 (1.1)	B (1)	–	–	(37)
	January 2011– December 2015, January 2019– December 2021	22/1,645 (1.3)	–	6/1,645 (0.4)	–	4/1,645 (0.2)	–	(4)
AIDS/HIV patients	July 2013– March 2017	6/155 (3.9)	*C. hominis* (2): IaA28R4 (1), IeA12G3T3 (1); *C. andersoni* (2): NA *C. meleagridis* (1): NA *C.parvum* (1): NA	3/155 (1.9)	B + C (3)	8/155 (5.2)	D (2); A, EbpC, I, Type IV, Peru11 and EbpD (one each)	(38)
Total		203/8,732 (2.3)	*C. hominis* (133): IaA14R4 (62); IdA19 (37); IbA19G2 (1), IdA14 (1), IaA18R4 (1), IgA14 (1); IaA28R4 (1), IeA12G3T3 (1); *C. andersoni* (36): NA; *C. meleagridis* (7): NA; *C. canis* (2): NA *C. felis* (2): NA; *C. parvum* (1): NA	245/6,588 (3.9)	AII (178); B (13); C (16); B + C (3)	53/2,625 (2.0)	Peru 11 (7); D (3); EbpA (2); EbpC (2); SH2 (3); A, EbpD, I, SH1, SH3–12, and TypeIV (one each)	

^*a*^ The bars denote negative results.

^*b*^ NA = not available.

*Cryptosporidium hominis* was the species found in the two positive samples. It has been detected in wastewater samples in Australia (39), Brazil and Peru (40), Japan (41), Switzerland and Germany (42), the United States (43), Tunisia (44), and China (28, 30). Finding *C. hominis* in wastewater is not surprising in that human intestinal pathogens are present in feces and therefore in wastewater. There are 49 species and >120 genotypes of *Cryptosporidium* identified (45). Of those, 19 species and four genotypes have been reported in humans (46). Cryptosporidiosis is caused by *C. hominis* and *C. parvum* in ~95% of infections worldwide (46). *C. hominis* is responsible for more infections than *C. parvum* in most industrialized nations and developing countries (47, 48). However, in China, *C. hominis* has the highest frequency (48.3%) in human cases (127/263) (10). *C. hominis* is also the predominant organism in frequently studied populations (children, diarrheal patients, and HIV/AIDS patients) in Shanghai, representing 65.5% of human cases of cryptosporidiosis (Table 3). We identified a rare subtype IdA14 based on sequence analysis of the *gp60* gene. Current epidemiological data indicate that human cryptosporidiosis cases due to subtype IdA14 have only been found in Australia (49), Sweden (50), and China (Xinjiang and Shanghai) (33, 51). This subtype has only been detected in one animal species (macaques) in China (52) suggesting a limited geographical distribution and narrow host range.

We identified assemblages A (*n* = 8) and C (*n* = 4) in 12 *G*. *duodenalis*-positive samples. Prior surveys reported that assemblages A and B were predominant with sporadic assemblages of C, F, and G (53). The similar results were observed in China (Table 1). *G. duodenalis* is known as a multispecies complex, composed of eight assemblages (A to H) with different host specificity (8). Among them, six assemblages have been found in humans (8). Human epidemiological data regarding *G. duodenalis* demonstrate that ~96% of human infections are caused by assemblages A and B, with assemblages C–F occasionally found (54). From the onset of molecular epidemiological studies of human *G. duodenalis* infection in China, beginning in 2000, a total of 285 human-derived *G. duodenalis* samples obtained from 11 studies have been analyzed molecularly (33–38, 54–59) (Table S1). We observed that assemblage A was most prevalent (74.7%) followed by assemblage B (18.6%) and assemblage C (6.7%). Assemblages A and B had a wider geographical distribution than assemblage C: A and B in six and four investigated areas, respectively, whereas C was found only in the investigated area Shanghai (Table S1). This was consistent with the genotyping of *G. duodenalis* cysts in wastewater samples. We found that the *G. duodenalis* assemblage A belonged to the anthroponotic sub-assemblage AII. Identification of the genetic variations in nucleotide sequences within assemblage A at the *bg*, *tpi*, and *gdh* loci led to the designation of three sub-assemblages AI, AII, and AIII having different host specificity (60). Sub-assemblages AI and AII have been commonly found in animals and humans, respectively, whereas sub-assemblage AIII has only been found in animals, mostly wildlife (61). In China, 96.1% (199/207) of human-derived isolates of *G. duedenalis* assemblage A belong to sub-assemblage AII including the study area in Shanghai (33–38, 54–59) (Table S1).

We identified genotype A in only one *E. bieneusi-*positive sample. There have been at least 819 genotypes recognized in humans and animals per sequence analysis of the ITS region of the rRNA gene, with at least 184 *E. bieneusi* genotypes recorded in humans (7). The genotypes D, A, and Type IV are the most common in humans infected with *E. bieneusi* according to one review (7). Genotype A is considered indicative of person-to-person transmission of *E. bieneusi* (61, 62). This genotype is primarily found in humans (7). Limited epidemiological data indicate that genotype A of *E. bieneusi* is found in animals—captive baboons (*n* = 1) in Kenya (63) and dogs (*n* = 1) in Spain (64). In China, genotype A was only found in humans—children (Xinjiang) (65) and HIV/AIDS patients in the investigated area of Shanghai (38). The genotyping results of *E. bieneusi* of local HIV/AIDS patients were consistent with those of the wastewater investigated in our study.

We found *Cryptosporidium* spp., *G. duodenalis,* and *E. bieneusi* in the influent and effluent wastewater of Shanghai treatment plants, indicating the necessity of better wastewater treatment and the importance of monitoring pathogens in effluents. Meanwhile, it was a reflection of the occurrence rate and genetic characterizations of the three intestinal pathogens from members of the community served by the wastewater treatment plant. Homology analysis of the three intestinal protozoa indicated that the person-to-person transmission possibly occurred in community members served by the wastewater treatment plant. This coincidental finding was observed in the occurrence rates and genotyping results of the three intestinal protozoa found in our study samples and the reports of previous studies, further suggesting that WBE can be used as an effective epidemic management tool within communities. However, this study has some limitations. There was only a small number of wastewater samples analyzed, and positive samples from either influents or effluents were small. This possibly resulted in the inconsistent time of occurrence of positive wastewater samples for each of the three intestinal protozoa from influents or effluents. To better understand the prevalence and genetic characterization of the pathogens that cause diarrhea in human populations, future WBE studies of intestinal protozoa should enlarge the number of wastewater samples and increase sampling frequency.

## MATERIALS AND METHODS

### Sample collection and procession

From July 2011 to June 2012, we collected 48 urban wastewater samples (24 from influents and 24 from effluents) from YPWTP in Shanghai, China. There are around 1,240,000 persons served by the Yangpu Wastewater Treatment Plant. Samples were collected semimonthly, including one influent and one effluent wastewater sample. Influent and effluent samples were collected at the inlet and the outlet of the treatment plant, respectively. According to the description of Feng et al. (29), grab samples of influent raw wastewater (600–800 mL) were collected and sieved to filter out large impurities and then centrifuged at 1,500 g for 10 min. According to the description of Montemayor et al. (66), method 1623 (USEPA 1999) was used to concentrate effluent samples (20 L each) by EnvirochekTM sampling capsules (Pall Gelman Laboratory, Ann Arbor, MI, USA). After centrifugation at 1,500 × *g* for 15 min (Eppendorf 5810; Eppendorf, Hamburg, Germany), approximately 200 mg of each sediment was used to extract genomic DNA for further molecular analysis.

### DNA extraction

Sample pellets were washed twice in phosphate-buffered saline (PBS, pH7.4) to extract genomic DNA using the Fast DNA SPIN Kit for Soil (BIO101, Carlsbad, CA, USA) according to the manufacturer’s instructions. DNA was eluted in 100 µL of AE buffer and stored at −20°C for subsequent analysis.

### PCR amplification

Each DNA sample was analyzed at least three times for each of the pathogens using 1 µL of the DNA templates in a 25 µL PCR reaction volume [2 × TransTaq-T PCR SuperMix (+dye)]. Following analysis using nested PCR amplification, secondary PCR products were separated in a 1.5% agarose gel electrophoresis and visualized on a GelDocTM EZ Imager (Bio-Rad, United States) using an ethidium bromide stain.

### Genotyping and subtyping of pathogens

We used nested PCR amplification to type pathogens. *Cryptosporidium* species were identified using a fragment (~830 bp) of the SSU rRNA gene (67). *Cryptosporidium*-positive DNA samples were further subtyped using 850 bp fragments of the *gp60* gene (68). To identify *G. duodenalis* assemblages and sub-assemblages, a 530 bp fragment of the *tpi* gene was amplified (69). *E. bieneusi* was identified and genotyped using a 390 bp fragment of the rRNA gene including 243 bp of the ITS region (70). The specific primers and reaction conditions of four target genes are summarized in Table S2.

### Sequence analysis

All secondary PCR products were sequenced using their respective PCR primers on an ABI PRISMTM 3730 DNA Analyser (Applied Biosystems, Carlsbad, CA, USA), using a BigDye Terminator v3.1 Cycle Sequencing kit (Applied Biosystems). Sequence accuracy was ensured by sequencing PCR products in both directions and by sequencing another two PCR products for DNA samples having novel nucleotide sequences (in comparison to those published in the GenBank database).

## Data Availability

Representative nucleotide sequences generated from the study were submitted to GenBank under accession numbers: KJ019853 to KJ019855 (Cryptosporidium) and KJ019857 to KJ019868 (*G. duodenalis*).

## SUPPLEMENTAL MATERIAL

Table S1 spectrum.04032-23-s0001.docx Distribution of *G. duodenalis* assemblage and sub-assemblage.

Table S2 spectrum.04032-23-s0002.docx Primers and reaction program of PCR amplification.

ASM does not own the copyrights to Supplemental Material that may be linked to, or accessed through, an article. The authors have granted ASM a non-exclusive, world-wide license to publish the Supplemental Material files. Please contact the corresponding author directly for reuse.
