# Supplementary material for: Wastewater-based intestinal protozoa monitoring in Shanghai, China
Source: Microbiol Spectr. 2024 Sep 24;12(11):e04032-23. doi: 10.1128/spectrum.04032-23 (PMC11540151; doi:10.1128/spectrum.04032-23)
Supplement: Table S1 — Distribution of G. duodenalis assemblage and sub-assemblage. [file spectrum.04032-23-s0001.docx]

**Table S1** Distribution of *G. duodenalis* assemblage and sub-assemblage in different populations in China.

| **Location** | **Population** | **Positive no.** | **Assemblage (n)** | | | **Sub-assemblage (n)** | | **Reference** |
| --- | --- | --- | --- | --- | --- | --- | --- | --- |
|  |  |  | **A** | **B** | **C** | **AI** | **AII** |  |
| Anhui | Patients | 8 | 4 | 4 | — | 0 | 4 | 55 |
| Hebei | Residents | 3 | 3 | — | — | 0 | 3 | 56 |
| Henan | Inpatients | 18 | 12 | 6 | — | 8 | 4 | 57 |
|  | Children | 14 | 4 | 8 | — | 0 | 2 | 58 |
| Hubei | Diarrhea children | 7 | 7 | — | — | 0 | 7 | 59 |
| Heilongjiang | Diarrhea patients | 24 | 5 | 19 | — | 0 | 1 | 54 |
| Shanghai | Children with various congenital or inherited diseases | 7 | 6 | — | — | 0 | 6 | 34 |
|  | Pediatric patients treated by the Departments of Endocrinology, Hematology, Neurology, and General Surgery | 4 | 2 | 2 | — | 0 | 2 |  |
|  | Children from unknown wards in Hospitals | 25 | 17 | 9 | — | 0 | 17 |  |
|  | Diarrhea patients | 17 | — | 1 | 16 | 0 | 0 | 36 |
|  | Diarrhea patients | 1 | — | 1 | — | 0 | 0 | 37 |
|  | Children | 153 | 153 | — | — | 0 | 153 | 35 |
|  | HIV/AIDS patients | 3 ^a^ | — | 3 | 3 | 0 | 0 | 38 |
| Total | | 296 | 213 | 53 | 19 | 8 | 199 |  |
| The bar denotes negative results.  ^a^ Mixed infection sample. | | | | | | | | |
