# Supplementary material for: Wastewater-based intestinal protozoa monitoring in Shanghai, China
Source: Microbiol Spectr. 2024 Sep 24;12(11):e04032-23. doi: 10.1128/spectrum.04032-23 (PMC11540151; doi:10.1128/spectrum.04032-23)
Supplement: Table S2 — Primers and reaction program of PCR amplification. [file spectrum.04032-23-s0002.docx]

Table S2 Primers and reaction program of PCR amplification for identifying Cryptosporidium and their gp60 subtypes, *G. duodenalis* and *E. bieneusi*.

| **Gene** | **Primer sequences (5′-3′)** | **Fragment size (bp)** | **Reaction condition** | **References** |
| --- | --- | --- | --- | --- |
| SSU *rRNA* | 18SU1: TTCTAGAGCTAATACATGCG | ~1350 | An initial hot start at 94°C for 3 min, 35 cycles of 94°C for 45 s, 55°C for 45 s, and 72°C for 60 s, a final extension step at 72°C for 7 min | 67 |
|  | 18SD1: CCCATTTCCTTCGAAACAGGA |  |  |  |
|  | 18SU2: GGAAGGGTTGTATTTATTAGATAAAG | ~850 |  |  |
|  | 18SD2: AAGGAGTAAGGAACAACCTCCA |  |  |  |
| *gp60* | AL3531: ATAGTCTCCGCTGTATTC | ~1000 | An initial hot start at 95°C for 3 min, 35 cycles of 94°C for 45 s, 50°C for 45 s, and 72°C for 60 s, a final extension step at 72°C for 10 min | 68 |
|  | AL3535: GGAAGGAACGATGTATCT |  |  |  |
|  | AL3532: TCCGCTGTATTCTCAGCC | ~850 |  |  |
|  | AL3534: GCAGAGGAACCAGCATC |  |  |  |
| *tpi* | AL3543: AAATIATGCCTGCTCGTCG | ~605 | An initial hot start at 94°C for 5 min, 35 cycles of 94°C for 45 s, 50°C for 45 s, and 72°C for 60 s, a final extension step at 72°C for 10 min | 69 |
|  | AL3546: CAAACCTTITCCGCAAACC |  |  |  |
|  | AL3544: CCCTTCATCGGIGGTAACTT | ~530 |  |  |
|  | AL3545: GTGGCCACCACICCCGTGCC |  |  |  |
| ITS region | EBITS3: GGTCATAGGGATGAAGAG | 435 | An initial hot start at 94°C for 5 min, 35 cycles of 94°C for  30 s, 57°C for 30 s, and 72°C for 40 s, a final extension step at 72°C for 10 min | 70 |
|  | EBITS4: TTCGAGTTCTTTCGCGCTC |  |  |  |
|  | EBITS1: GCTCTGAATATCTATGGCT | 390 | An initial hot start at 94°C for 5 min, 30 cycles of 94°C for 30 s, 55°C for 30 s, and 72°C for 40 s, a final extension step at 72°C for 10 min |  |
|  | EBITS2.4: ATCGCCGACGGATCCAAGTG |  |  |  |
